# Reduced spread of influenza and other respiratory viral infections during the COVID-19 pandemic in southern Puerto Rico

**DOI:** 10.1371/journal.pone.0266095

**Published:** 2022-04-27

**Authors:** Talia M. Quandelacy, Laura E. Adams, Jorge Munoz, Gilberto A. Santiago, Sarah Kada, Michael A. Johansson, Luisa I. Alvarado, Vanessa Rivera-Amill, Gabriela Paz–Bailey

**Affiliations:** 1 Dengue Branch, Centers for Disease Control and Prevention, San Juan, Puerto Rico; 2 Department of Epidemiology, University of Colorado Anschutz Medical Campus, Aurora, Colorado, United States of America; 3 Ponce Health Sciences University, Ponce, Puerto Rico; Freelance Consultant, Myanmar, MYANMAR

## Abstract

**Introduction:**

Impacts of COVID-19 mitigation measures on seasonal respiratory viruses is unknown in sub-tropical climates.

**Methods:**

We compared weekly testing and test-positivity of respiratory infections in the 2019–2020 respiratory season to the 2012–2018 seasons in southern Puerto Rico using Wilcoxon signed rank tests.

**Results:**

Compared to the average for the 2012–2018 seasons, test-positivity was significantly lower for Influenza A (p<0.001) & B (p<0.001), respiratory syncytial virus (RSV) (p<0.01), respiratory adenovirus (AdV) (p<0.05), and other respiratory viruses (p<0.001) following March 2020 COVID-19 stay at home orders.

**Conclusions:**

Mitigation measures and behavioral social distancing choices may have reduced respiratory viral spread in southern Puerto Rico.

## Introduction

Since the emergence of severe acute respiratory syndrome coronavirus 2 (SARS-CoV-2) in December 2019 [1], there has been concern of co-circulation of influenza and SARS-CoV-2 in the United States for the winter months of 2020–2021 [2]. Overall declines in influenza infections from March to July 2020 were observed in the Southern Hemisphere [3], and these reductions were attributed to the public’s increased awareness of preventative measures such as hand hygiene and mask wearing, and population-level mitigation efforts like social distancing. Declines in other respiratory pathogens, such as respiratory syncytial virus (RSV) and adenovirus, have similarly been observed globally [4, 5].

In the United States (US), influenza trends have declined and remained historically low since March 2020 [6, 7], suggesting that Coronavirus Disease-2019 (COVID-19) mitigation measures are impacting influenza virus transmission [3]. Respiratory syncytial virus (RSV) was estimated to have declined by 20% in the US since the start of the COVID-19 pandemic [8], though increased circulation of RSV and respiratory adenoviruses have been reported in the US since early spring 2021 [6, 9].

In tropical locations, where respiratory virus seasonality is more varied, changes in respiratory viral transmission is also a question of interest. An evaluation of nine tropical counties in Asia found shorter circulation of respiratory infections as well as reduced circulation, possibly due to layered non-pharmaceutical interventions implemented since 2020 [10]. There is limited information on the impact of NPIs implemented during the COVID-19 pandemic on respiratory infections in the Caribbean [11, 12]. Despite the higher temperatures and humid climate in Puerto Rico, pre-COVID-19 pandemic influenza trends on the island were similar to those in the continental United States, with peak activity occurring in the months of December and January, but often circulating for longer periods than typically observed in the continental US [13]. A study of pediatric respiratory infections found RSV exhibited similar trends in seasonality to influenza, and AdV having stronger seasonal peaks in the spring and early summer months [14].

To assess changes in respiratory viral activity since the weeks following the initial COVID-19 pandemic mitigation measures in Puerto Rico and in continuation to long term pandemic measures, we compared the weekly trends of influenza and other respiratory viral infections in southern Puerto Rico from September 29, 2019 to September 21, 2020 (surveillance weeks 40 of 2019–39 of 2020) and the weekly trends before and after island-wide mitigation measures implemented on March 15, 2020 (week 12) to the average of corresponding weeks from the 2012–2013 to 2018–2019 seasons.

## Materials and methods

### Data

The Sentinel Enhanced Dengue Surveillance System (SEDSS) has been previously described [13, 14]. Briefly, we collected data from the Sentinel Enhanced Dengue Surveillance System (SEDSS) in the southern region of Puerto Rico, a surveillance system that has been monitoring acute febrile illness (AFI) trends among three sentinel hospitals in Ponce, Puerto Rico and surrounding municipalities since May 2012 [13, 14], and tested for the presence of arboviral and respiratory virus etiologies. Individuals seeking medical care at the three participating hospitals are screened for AFI, defined as reporting fever (>37.5°C) at the time of visit or self-reported fever in the past seven days. Individuals fitting these criteria are invited to participate in AFI surveillance. Enrolled participants complete a questionnaire and nasal swab samples and a serological sample are collected. Nasal swab samples are tested by real time reverse transcriptase polymerase chain reaction (RT-PCR) for several arboviral and respiratory viruses including Influenza (A & B), RSV, AdV, and other respiratory viruses (i.e., human parainfluenza virus types 1 & 3 and human metapneumovirus), as previously described [13, 15–17].

All enrolled patients or legally authorized representatives provided written informed consent prior to enrollment in accordance with the Puerto Rico Law (Article 13, Section 13, Regulation 7617 of the Office of Patient Ombudsman, Act #194) as previously described [18]. The Ponce Medical School Foundation and the US Centers for Disease Control and Prevention (CDC) ethics committees reviewed and approved the SEDSS study protocol.

**Fig 1:**
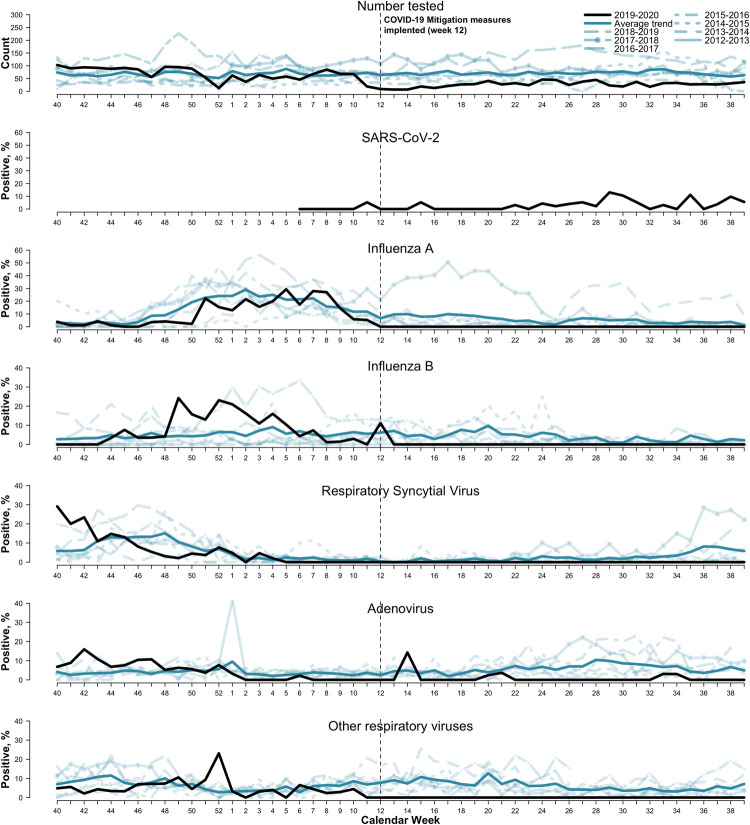
Number tested and test positivity for SARS-CoV-2, influenza A & B, respiratory syncytial virus (RSV), respiratory adenovirus (AdV), and other respiratory viruses (human metapneumovirus and human parainfluenza virus types 1 & 3) from the 2012–2019 respiratory seasons, southern Puerto Rico, SEDSS. Line types indicate the different respiratory seasons. The trend the average weekly percent test-positive from surveillance week 40 to 39 across the 2012–13 to 2018–19 seasons. The vertical dashed line corresponds to March 15, 2020 (week 12), when island-wide COVID-19 mitigation measures were implemented.

Data were available from surveillance weeks 40 of 2019 to 39 of 2020 (i.e., 2019–20 season). Using the weekly number of cases from surveillance week 40 to 39 for each season from 2012 to 2018, we averaged the number of cases in each week across the seven seasons to generate the average weekly number of cases for weeks 40 to 39, hereafter referred to as the 2012–18 average season. We visually compared the weekly time-series trends of the 2019–20 season to that of the 2012–18 average season (Fig 1).

Using additional data from the 2020–2021 season (surveillance week 40 of 2020 to week 39 of 2021), we visually compared the respiratory infection trends of the 2019–2020 season to trends observed in the 2020–2021 season to assess the similarity of these since the start of the pandemic (S1 File). Similarly, we evaluated average trends of dengue over the 2012–18 seasons and compared the average seasonal trend to the weekly time-series of the 2019–20 and 2020–21 season (S1 File).

### Statistical analyses

Non-parametric Wilcoxon signed rank tests were used to assess pairwise differences in weekly number of participants tested and the mean weekly test positivity of each respiratory virus in the 2019–20 season to the 2012–18 average season. Differences were examined over three periods using one- or two-sided tests to determine: 1) if the weekly number tested and viral test-positivity in the entire 2019–20 season (i.e., weeks 40 to 39) were the same as the corresponding weeks of the 2012–18 average season, with no assumption on the direction of the difference (i.e., two-sided test); 2) if the weekly test-positivity prior to mitigation measures implemented on March 15, 2020 (i.e., weeks 40 to 11) was *lower* than the corresponding weeks of the 2012–18 average season (i.e., one-sided test), and 3) if the weekly test-positivity in the weeks following mitigation measures (weeks 12 to 39) was *lower* than those weeks in the 2012–2018 average season (i.e., one-sided test). Statistical significance was assessed using a 5% significance level, and analyses were conducted using R version 3.6.3.

## Results

On March 11, 2020, Puerto Rico confirmed its first cases of SARS-CoV-2 in Puerto Rico [19]. The first case of SARS-CoV-2 confirmed in the SEDSS system in southern Puerto Rico was during the week of March 9^th^, 2020 (week 11 of 2020). Since then, 132 SARS-CoV-2 cases have been laboratory confirmed between March 2020 and September 2021 in the SEDSS system in Southern Puerto Rico. During the 2019–2020 season, 2,439 people presented with acute febrile illness and were tested for respiratory viruses within SEDSS (Table 1) and 1,803 people were tested during the 2020–2021 season. Overall, the number of tests performed during the 2019–2020 season was lower compared to the 2012–18 average season (p<0.001; Table 1). Weekly testing was not significantly lower during weeks 40 to 11 (*P* = 0.69, Table 1), but was significantly lower in the weeks following the island-wide mitigation measures (weeks 12 to 39) (*P*<0.001), with numbers down to ~27 samples per week, potentially limiting detection with low levels of virus circulation.

**Table 1:** Number tested and test-positivity of influenza and other viral respiratory pathogens in the 2019–20 season compared to the average across the 2012–18 seasons, overall and before and after implementation of COVID-19 mitigation measures in southern Puerto Rico, SEDSS.

	A) Entire season (Weeks 40 to 39)			B) Period before COVID-19 mitigation measures (Weeks 40 to 11)			C) Period after COVID-19 mitigation measures (Weeks 12 to 39)		
	2019–20	2012–18 average season*	*P*-value^†^	2019–20	2012–18 average season*	*P*-value^‡^	2019–20	2012–18 average season*	*P*-value^§^
**Number tested**	2,439	3,604	P<0.001	1,674	1,624	P = 0.69	765	1,980	P<0.001
**No. Influenza A test-positivity (%)**	154 (6%)	387 (11%)	P<0.001	154 (9%)	250 (15%)	P<0.01	0 (0%)	137 (7%)	P<0.001
**No. Influenza B test-positivity (%)**	118 (5%)	165 (5%)	P = 0.05	117 (7%)	98 (6%)	P = 0.91	1 (<1%)	67 (2%)	P<0.001
**No. RSV test-positivity (%)**	134 (5%)	133 (4%)	P<0.001	134 (8%)	95 (6%)	P = 0.13	0 (0%)	38 (2%)	P<0.001
**No. AdV test-positivity (%)**	95 (4%)	159 (4%)	P<0.001	90 (5%)	60 (4%)	P = 0.62	5 (1%)	99 (5%)	P<0.05
**No. Other respiratory viruses test-positivity (%)**	80 (3%)	229 (6%)	P<0.001	80 (5%)	112 (7%)	P = 0.02	0 (0%)	117 (6%)	P<0.001

Other respiratory viruses are laboratory-confirmed cases of human metapneumovirus and human parainfluenza virus types 1 & 3.

*The average trend is the average weekly percent test-positive from surveillance week 40 to 39 across the 2012–13 to 2018–19 seasons.

^†^Two-sided p-value for Wilcoxon signed rank test assessed the pairwise difference between weekly outcomes (weekly number of tests and weekly test-positivity) across the entire 2019–2020 season compared and the average across the 2012–18 seasons (weeks 40 to 39).

^‡^One-sided p-value for Wilcoxon signed rank test assessing if weekly outcomes were lower in the 2019–20 season (comparing weeks 40 to 11, i.e., before COVID-19 mitigation measures) compared to the average across the 2012–18 seasons.

^§^One-sided p-value for Wilcoxon signed rank test assessing if weekly outcomes were lower in the 2019–20 season (weeks 12 to 39, i.e., after COVID-19 mitigation measures) compared to the average across the 2012–18 seasons. Abbreviations: RSV, respiratory syncytial virus; AdV, respiratory Adenovirus.

Testing for SARS-CoV-2 in SEDSS began on February 3, 2020 (surveillance week 6 of 2020), and as of September 9, 2020 (week 39) (Fig 1), 21 cases had been confirmed within this surveillance system. For Influenza A and other respiratory viruses, test-positivity in 2019–2020 was significantly different from the average weekly test-positivity across the 2012–2018 seasons (*P*<0.001 and P<0.001, respectively), and was lower both prior to (*P*<0.01 and P = 0.02, respectively) and following COVID-19 mitigation measures (*P*<0.001 and P<0.001, respectively). Weekly test-positivity of influenza B was not different over the 2019–20 season compared to the 2012–18 average season (*P* = 0.05) nor prior to March 15, 2020 (P = 0.91), but was significantly lower in weeks 12–39 (*P*<0.001). Similar decreases in test-positivity were observed for RSV and AdV, with similar test-positivity for these viruses to the 2012–18 average season in weeks 40–11 (RSV: *P* = 0.13 and AdV: *P* = 0.62), but significantly lower test-positivity in weeks 12–39 (RSV: *P*<0.001 and AdV: *P*<0.05; Fig 1, Table 1).

Weekly trends were similar when comparing the 2019–20 and 2020–21 seasons for most respiratory infections (S1 Fig in S1 File). In the 2020–2021 season, some in test-positivity were observed for RSV, and other respiratory viruses. Reduced test-positivity for dengue showed there was also an impact on testing and test-positivity of other non-respiratory viruses, during the 2019–20 and 2020–21 seasons compared to previous seasons (S2 Fig in S1 File).

## Discussion

In southern Puerto Rico, test positivity for influenza and other respiratory viral infections began decreasing below the average for previous seasons in late February and remained low after island-wide mitigation measures were implemented on March 15, 2020 (Fig 1). Seasonal declines of influenza positivity in SEDSS typically begin in the weeks between March and May, as observed in the average 2012–2018 season. Cases of influenza A began declining in the weeks leading up to March 15, 2020, suggesting that, at least with respect to influenza A, changes in testing or individual’s social distancing behavior taken ahead of the island-wide measures may have had an early impact on the number of influenza cases reported and are similar to decreases observed in the US [3, 20].

The notable change in the rate of decline in cases of influenza A & B, RSV, AdV and other respiratory viruses after March 15, 2020 suggests this earlier decline may correspond to the initial stay at home order and COVID-19 mitigation measures. Similar declines in influenza cases around March 15, 2020 have also been reported across Puerto Rico [21]. These declines contrast with the pattern observed with SARS-CoV-2, where the percent SARS-CoV-2 test positivity increased from week 23 of 2020 and onward. It is unclear how much the reductions in test-positivity and testing are impacted by fewer people seeking medical care or other secular/ecological trends, as the system may have been limited in detecting positive cases in the smaller sample size with very low levels of virus circulation. Still, spikes in AdV test-positivity indicate that the surveillance system was active and could detect respiratory infections after the initial island-wide stay-at-home order and mitigation measures were in place.

Other factors may have further impacted the transmission of respiratory infections during the 2019–20 season in Puerto Rico. SEDSS serves as one of the only ILI surveillance systems on the island [22, 23] but these reductions in weekly respiratory infections correspond with reported declines observed in influenza observed across the island, as previously mentioned. At the start of 2020, influenza A/H1N1(pdm09) was the predominantly circulating strain with influenza B/Victoria also circulating [24] and the influenza vaccine composition for the 2019–2020 was considered well matched to the circulating influenza A/H1N1(pdm09) and influenza B/Victoria strains) [25]. The vaccine uptake rate in Puerto Rico in March 2020 ranged from 32–41% for children and 14–27% for adults [26] and estimates ranged from 24–31% for children and 16–36% adults in the 2020–21 season [27]. These rates are slightly higher than the historical average influenza vaccination rate of 18% [28] and may have had some impact on further reducing influenza infections among some age-groups.

As the transmission dynamics of influenza and other respiratory viruses in Puerto Rico are synchronized with mainland US [13], drops in connectivity due to the disruption of air traffic, decreases in family visits, tourism and cancellation of large gatherings as part of suggested COVID-19 guidelines may have further affected the circulation of these respiratory viral pathogens. While there were no travel border restrictions in Puerto Rico outside of those related to limits on travel to the US from other countries [29], reductions of cruise ships [30] and international travel to Puerto Rico may have contributed to a reduction of imported respiratory infections into the island. It is unclear how changes in air travel from the continental US to Puerto Rico may have impacted trends of the respiratory.

Our analysis had some limitations. Dramatic decreases in the number of tests samples were observed in our surveillance system, a situation observed across the region [11]. This could be due to a decrease in primary case detection during the period of mitigation measures and later or a decrease in testing for non-SARS-CoV-2 data. The small sample size of the weekly number of tests during the pandemic likely limited the ability of SEDSS to detect low level circulation, and given the geographic region that this system captures, our observed trends could be underestimates of respiratory infections.

Other countries in the Southern Hemisphere and elsewhere have reported lower influenza activity during the COVID-19 pandemic [3]. Similar declines in influenza cases were reported across China, Hong Kong, Taiwan, Japan, South Korea, Singapore, and Turkey [31–35]. These declines have been broadly attributed to population-wide preventative measures, including handwashing, face mask wearing and social distancing. Previous to COVID-19, significant events that restrict human mobility, such as major winter storms, have shown reductions in transmission of influenza as well as other respiratory infections [36] Preventative measures such as mask wearing and social distancing have become and remain important mitigation measures for reducing transmission of SARS-CoV-2 [37], and growing evidence suggests these may have a greater impact on reducing the spread of influenza [38, 39], RSV, AdV and other respiratory infections even in warmer climates.

## Supporting information

S1 File Supporting information for “Reduced spread of influenza and other respiratory viral infections during the COVID-19 pandemic in southern Puerto Rico”. This supplement contains additional figures.
(DOCX)
