# Supplementary material for: Reduced spread of influenza and other respiratory viral infections during the COVID-19 pandemic in southern Puerto Rico
Source: PLoS One. 2022 Apr 27;17(4):e0266095. doi: 10.1371/journal.pone.0266095 (PMC9045654; doi:10.1371/journal.pone.0266095)
Supplement: S1 File — This supplement contains additional figures. (DOCX) [file pone.0266095.s001.docx]

**Supplementary Information**
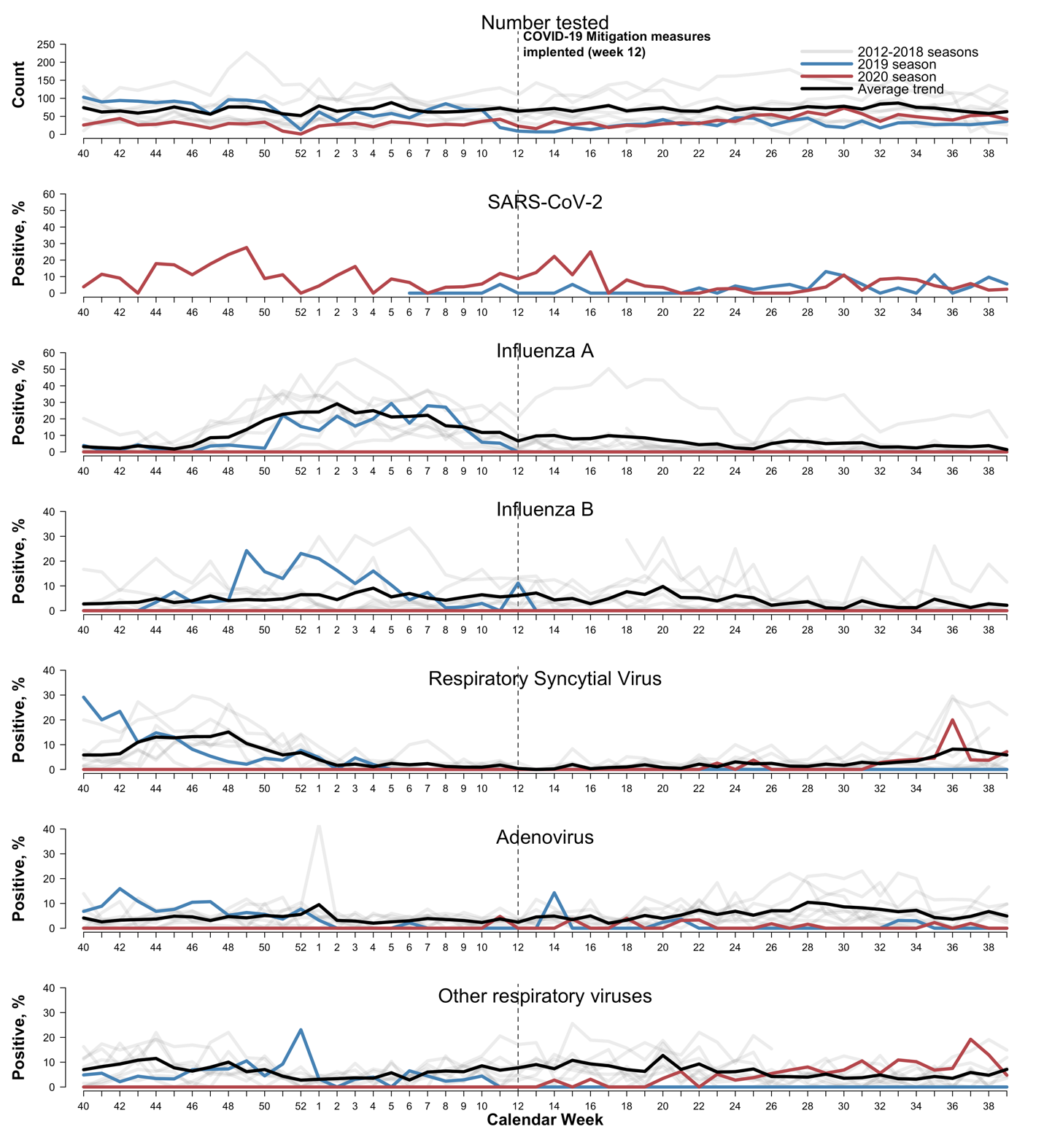


**Supplementary Figure 1. Number tested and test positivity for SARS-CoV-2, influenza A & B, respiratory syncytial virus (RSV), respiratory adenovirus (AdV), and other respiratory viruses (human metapneumovirus and human parainfluenza virus types 1 & 3) from the 2012-2020 respiratory seasons, southern Puerto Rico, SEDSS.** Transparent grey lines reflect weekly trends for the 2012-2018 seasons. The solid black line reflects the average seasonal trend from the 2012-2019 season. The weekly trends of the 2019-20 season and 2020-21 season are shown in solid blue and red lines. The vertical dashed line corresponds to March 15, 2020 when island-wide COVID-19 mitigation measures were implemented.

**
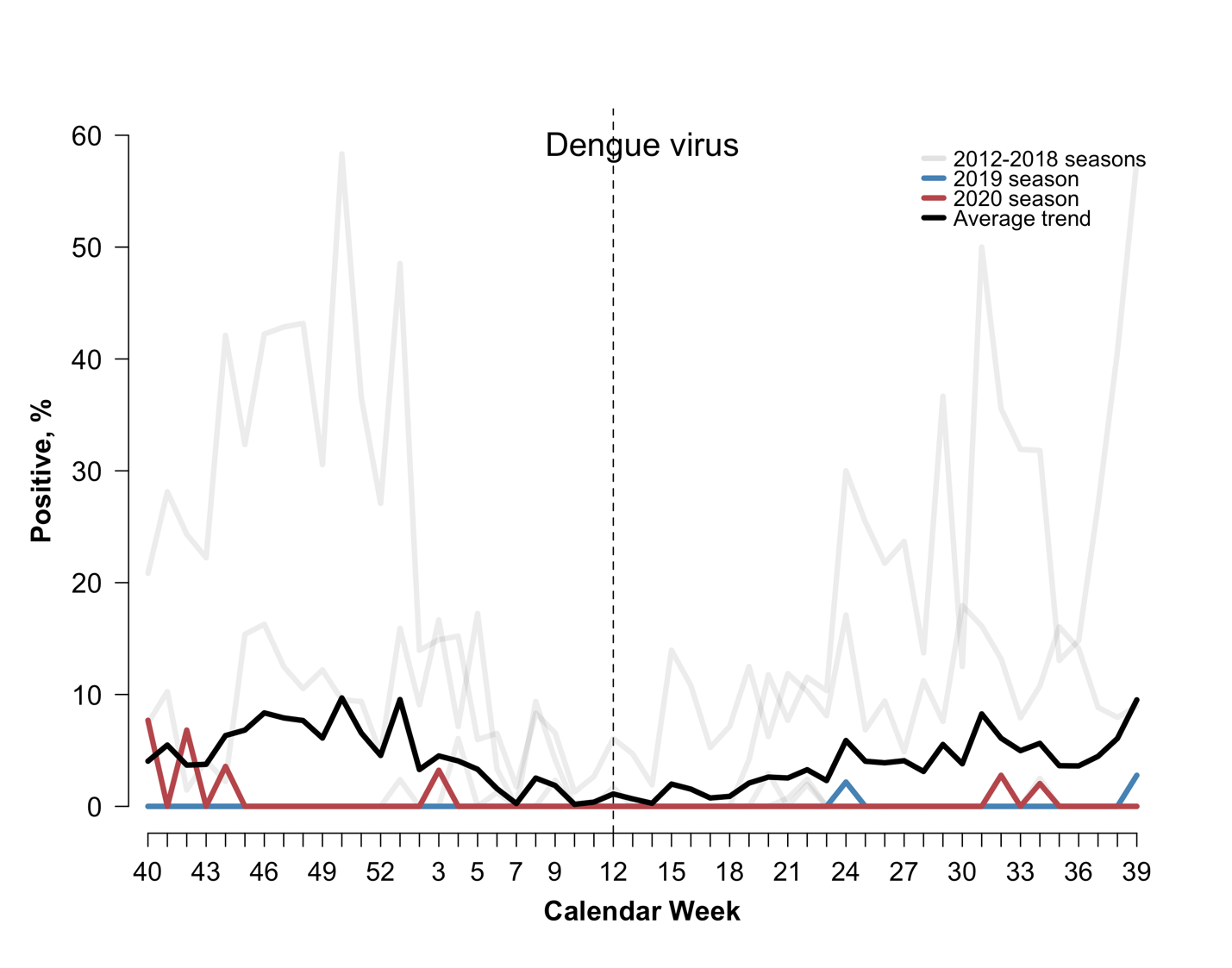
**

**Supplementary Figure 2. The test positivity for dengue virus from the 2012-2020 respiratory seasons, southern Puerto Rico, SEDSS.** Transparent grey lines reflect weekly trends for the 2012-2018 seasons. The solid black line reflects the average seasonal trend from the 2012-2019 season. The weekly trends of the 2019-20 season and 2020-21 season are shown in solid blue and red lines. The vertical dashed line corresponds to March 15, 2020 when island-wide COVID-19 mitigation measures were implemented.
